# *Schistosoma mansoni* Infection Is Impacted by Malnutrition

**DOI:** 10.3389/fmicb.2021.635843

**Published:** 2021-03-19

**Authors:** Poliane Silva Maciel, Ricardo Gonçalves, Lis Ribeiro do Valle Antonelli, Cristina Toscano Fonseca

**Affiliations:** ^1^Laboratório de Biologia e Imunologia de Doenças Infecciosas e Parasitárias, Instituto René Rachou, Fundação Oswaldo Cruz, Belo Horizonte, Brazil; ^2^Laboratório de Biologia de Monócitos e Macrófagos, Departamento de Patologia Geral, Instituto de Ciências Biológicas, Universidade Federal de Minas Gerais, Belo Horizonte, Brazil

**Keywords:** schistosomiasis, *S. mansoni*, malnutrition, low-protein diet, low-fat diet

## Abstract

Schistosomiasis remains one of the most important neglected tropical diseases in the world. It mainly affects developing countries, where it often coexists with malnutrition. Despite this, few studies have investigated the relationship between schistosomiasis and malnutrition. Herein, we evaluate the impact of malnutrition on experimental *S. mansoni* infection. Mice were divided into 5 groups: Control (Ctrl) diet (14% protein and 10% lipids), low-protein 3% (LP 3%), low-protein 8% (LP 8%), low-fat 2.5% (LF 2.5%), and low-fat 5% (LF 5%). Mice were fed with their respective diets and were infected when a difference of approximately 20% in the body weight between mice from any experimental group and mice from the control group was achieved. Nutritional, parasitological, and immunological parameters were assessed either just before infection and/or approximately 50 days later before mice were perfused. Our results showed that the 3% low-protein diet was the only one capable of establishing malnutrition in mice. Mice fed with this diet showed: (i) significant reduction in body weight and serum albumin levels before infection, (ii) decreased levels of all biochemical parameters evaluated before perfusion, (iii) decreased numbers of schistosome eggs trapped in intestines and impaired parasite fecundity, (iv) a delay in the granuloma development with a smaller granuloma area, and (v) reduced levels of IL-4 and IFN-γ in the liver. Our findings demonstrate that low protein supply leads to malnutrition in mice and impacts the cytokine milieu in the liver and granuloma formation. Additionally, the establishment of our murine malnutrition model will enable future studies aiming to better understand the complex relationships between nutrition, immune responses, and infection outcome.

## Introduction

Schistosomiasis is a neglected tropical disease caused by parasites of the genus *Schistosoma* that remains as a major public health problem worldwide, being considered a disease of poverty (Ross et al., 2002; Gryseels et al., 2006; Colley et al., 2014; McManus et al., 2018). Approximately 800 million people are at risk of infection and 250 million are affected with schistosomiasis in 78 countries around the world, especially in the Middle East, South America, Southeast Asia and sub-Saharan Africa (WHO, 2020). The disease results in considerable human morbidity and mortality, notably in sub-Saharan Africa where more than 90% of all infections occur. The Global Burden of Disease (GBD) study of 2016 attributed 1.9 million disability-adjusted life years (DALYs) due to schistosomiasis (Gbd 2016 DALYs Collaborators, 2017).

Similar to schistosomiasis, malnutrition remains an important public health problem in developing countries. Protein energy malnutrition is the most frequent form of malnutrition, and globally affects 820 million people, including over 150 million children under 5 years of age, mainly in developing countries (FAO, 2019). Malnutrition is considered to be an important cause of immunodeficiency, predisposing the malnourished to a variety of different infectious diseases (Katona and Katona-Apte, 2008; Rodriguez-Morales et al., 2016). While malnutrition increases susceptibility to infection, infections aggravate malnutrition by decreasing appetite and increasing demand for nutrients, causing a vicious cycle (Katona and Katona-Apte, 2008; Bapat et al., 2015).

Malnutrition and intestinal parasitism share a similar geographic distribution, with individuals possibly experiencing both conditions. Thereby, schistosomiasis is commonly found where malnutrition is also prevalent (Coutinho et al., 1997; Atinmo et al., 2009; Barros et al., 2014; Mekonnen et al., 2014; Papier et al., 2014). Although there is evidence that malnutrition is one of the major factors influencing the outcome of schistosomiasis and *vice versa*, few studies have assessed the relationship between both conditions (Magalhães et al., 1986; Stephenson, 1994; Parraga et al., 1996; Coutinho et al., 1997, 2010; Barros et al., 2014).

In this context, the use of laboratory animals has been employed to understand how different degrees of malnutrition affect the host immune response, the susceptibility to infections, and pathogenesis (Borelli et al., 2007; Komatsu et al., 2007; Fock et al., 2008; Malafaia et al., 2009; Taylor et al., 2013). Most experimental models of malnutrition are limited to protein restriction (2–8% protein) (Anstead et al., 2001; Fock et al., 2007; Malafaia et al., 2009). Furthermore, in the case of schistosomiasis, there are no studies using the diet formulation proposed by the American Institute of Nutrition (AIN) (Reeves et al., 1993), a widely used diet in experimental nutrition studies (Borelli et al., 2007; Komatsu et al., 2007; Fock et al., 2008; Malafaia et al., 2009; Taylor et al., 2013). The use of this formulation attempts to standardize the food offered to animals in order to ensure reproducibility of biological research (Reeves et al., 1993; Pellizzon, 2016). Moreover, there are no studies that evaluate the effects of a low-fat diet on *S. mansoni* infection, an important macronutrient that the parasite depends on from its host (Meyer et al., 1970).

In this work, we used a murine model to address the impact of malnutrition on *S. mansoni* infection. We evaluated the impact of two isocaloric diets supplying distinct levels of protein, and two diets supplying distinct levels of lipids, on the infection of C57BL/6 mice with *S. mansoni*. Our findings demonstrate that only the 3% low protein diet altered *S. mansoni* infection. Mice that received this diet showed reduced liver pathology with smaller granuloma size, a delay in granuloma development, and a reduced number of schistosome eggs trapped in the intestine, reflecting a reduction in parasite fecundity. Distinct from the other groups, infection in LP 3%-fed mice did not increase the levels of TNF, which may be associated with the changes observed in liver pathology in this experimental group.

## Materials and Methods

### Mice and Diets

Female C57BL/6 mice (7–8 weeks old) were obtained from the Institute René Rachou (IRR)/FIOCRUZ (Fundação Oswaldo Cruz) animal facility. After weaning, mice were fed with the commercial diet CR1 (Nuvilab^®^, São Paulo, Brazil) until the beginning of the experiments. Mice were divided into 5 groups (12 animals/group) fed with the following experimental diets (Prag Soluções Biociências^®^, São Paulo, Brazil): Control (Ctrl): the standard diet AIN-93M (Reeves et al., 1993), containing 14 and 10% of protein and lipids, respectively; Low-protein (LP) 3 and 8%: a modified AIN-93M diet containing 3 or 8% protein, respectively; Low-fat (LF) 2.5 and 5%: a modified AIN-93M diet containing 2.5 or 5% lipids, respectively. All diets were isocaloric. The control and low-protein diets were identical, except that the casein removed from each of the formulations of the two low-protein diets was substituted by the same mass of corn starch. The soy oil removed from the low-fat diets was also replaced by corn starch. The energy value of both types of restricted diet (i.e., low-protein and low-fat) was slightly lower than that of the control diet. The daily food and protein consumption was determined by weighting the diets once a week. Mice body weights were assessed 3 times per week. Sample size calculation was performed using a 90% test power, a significance level of 0.05%, a standard deviation of 30 and a 40% expected difference between groups. All procedures on mice were authorized by the Ethics Committee of Animal Use of FIOCRUZ (LW2/18).

### Experimental Infection

A difference of 20% in the body weight between any experimental group and the control group was used to determine the time of the infection. *Schistosoma mansoni* LE strain cercariae were obtained by exposing infected *Biomphalaria glabrata* snails to light for 1–2 h to induce shedding. The mice were challenged through percutaneous exposure of shaved abdominal skin for 1 h to water containing approximately 100 cercariae according to Smithers and Terry (1965).

### Worm Burden Recovery and Number of Eggs in Gut and Liver

Mice were euthanized 50 days after infection and perfusion was performed to collect adult worms from the mesentery and liver as described by Pellegrino and Siqueira (1956). Intestine and liver were then harvested, weighed and digested with 10% KOH overnight at room temperature. The eggs were resuspended in 1 mL of saline after centrifugation at 900 *g* for 10 min. Three aliquots of 10 uL each from the suspension containing the parasite eggs was applied to glass slides, and the number of eggs per suspension was determined using a light microscope. Worm fecundity was evaluated as the ratio between the number of eggs per gram of intestine and the number of adult worm pairs. One centimeter of the terminal ileum was obtained from each mouse, the fecal content was removed, and then the intestine was pressed between plastic slides. Approximately 100 eggs were evaluated with regard to their stage of embryonic development (Pellegrinot and Faria, 1965). The percentage of immature eggs from the first, second, third and fourth stages, as well as those of mature and dead eggs, was determined.

### Measurement of Hepatic Granuloma Area

Fifty days post-infection, liver samples were taken from the central part of the left lateral lobe to assess granuloma formation. Liver samples were fixed in 4% formaldehyde in phosphate buffered saline (PBS), embedded in paraffin, and the histological sections obtained using microtome were stained with hematoxylin-eosin (HE). For the measurement of the hepatic granuloma area, images of all the granulomas in the exudative-productive stage (57–100 granulomas) for each group (5–10 granuloma from each mouse, 10–12 mice per group) were obtained using a camera attached to a light microscope (10 × objective lens). Only granulomas containing a single well-defined egg at the exudative-productive stage were selected from each liver section. The granuloma area (μm^2^) was measured using the AxioVision version 4.8 image analysis software (Carl Zeiss MicroImaging GmbH, Germany).

### Granuloma Classification

The developmental stages of *S. mansoni* granuloma formation were classified as previously described (Lenzi et al., 1998; Amaral et al., 2017). The following developmental stages of the granulomas were analyzed: exudative (E), the presence of an infiltrate of inflammatory cells in process of organization around the egg; exudative-productive (EP), characterized by organized infiltrate with circumferential aspect, showing a rich structure of collagen fibers and inflammatory cells concentrated in the periphery; and productive (P), with a thick layer of collagen fibers between the egg and a reduced number of inflammatory cells. A total of 100 granulomas from the Ctrl group, and 80 granulomas from the LP 3% group, were recorded (∼10 granulomas per mouse).

### Biochemical Parameter Evaluation

Hemoglobin, total protein and albumin levels were measured in blood samples taken from mice on the day (i) on which they initiated their experimental dietary regimen (day zero), (ii) before infection with *S. mansoni* (BI; 7 weeks after starting their experimental diets), and (iii) before perfusion (BP; 7 weeks after infection). Hemoglobin concentration was determined immediately after collection of blood samples using a commercial kit (Labtest Kit, Cat. 43, Lagoa Santa, MG, Brazil). Serum was separated by blood centrifugation, and total protein and albumin levels were determined using a commercial kit (Labtest Kit, Cat. 99 and 19, Lagoa Santa, MG, Brazil, respectively).

### Cytokine Analysis

The cytokine profile induced by different experimental diets was assessed using serum samples obtained from mice on the day they initiated their experimental dietary regimen (day zero), before infection (BI) and before perfusion (BP), and in the liver samples at BP only. One hundred milligrams of liver were homogenized in 1 mL PBS containing an antiprotease cocktail (0.1 mM phenylmethylsulfonyl fluoride, 0.1 mM benzethonium chloride, 10 mM EDTA, and 20 KI aprotinin A) and 0.05% Tween 20. Samples were then centrifuged for 10 min at 3,000 *g* and cytokine levels were determined using 25 μL of each supernatant. Cytokine measurement was performed using Cytometric Bead Array anti-mouse CBA Th1/Th2/Th17 Kit (BD Pharmingen, United States) according to the manufacturer’s protocol. Data acquisition was performed using a FACSVerse flow cytometer (BD, United States), and analyzed using the FCAP Array Software (Becton Dickinson).

### Statistical Analysis

GraphPad Prism 8.0 (Graph-Pad Software, San Diego, CA, United States) was used to perform the statistical analysis. Initially, the data were submitted to the Shapiro-Wilk normality test, followed by the Brown-Forsythe test to assess equality of variance. The results were analyzed using the non-parametric Mann-Whitney test, pair-by-pair, when the values of each group did not show equality of variance. In these cases, the alpha value was adjusted according to the number of comparisons made between the groups, and a 99% confidence level was applied. When the values presented equality of variance, statistical analysis was performed using either one-way ANOVA or the Kruskal-Wallis test, followed by either Tukey or Dunn’s multiple comparison tests, for parametric and non-parametric data, respectively. In these cases, a confidence level of 95% was applied. GraphPad Prism 8.0, taking into account the entire family of comparisons, automatically adjusted the p values.

## Results

### Low Protein 3% Diet Caused Malnutrition in Mice

**FIGURE 1:**
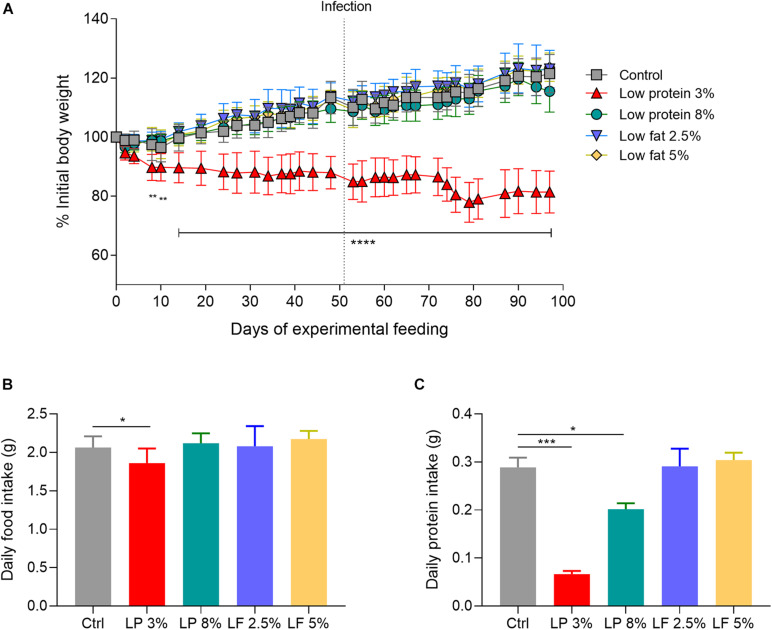
Alterations in the body weight of mice fed with different experimental diets. Mice received different restricted diets: Control (Ctrl), Low protein 3 and 8% (LP 3% and LP 8%, respectively) and Low fat 2.5 and 5% (LF 2.5% and LF 5%, respectively). Body weights **(A)** were measured three times a week. Dotted line indicates the time point when the mice were infected with ∼100 cercariae of *S. mansoni*. Food **(B)** and protein intake **(C)** were measured daily, and are represented as per day/per mouse in grams. Values are presented as mean ± *SD* in each graph. Results are representative of two independent experiments (*n* = 12 mice per group). Significant differences were determined by Two-way ANOVA followed by Tukey’s multiple comparison test **(A)** or one-way ANOVA followed by Holm-Sidak’s **(B,C)** (*p ≤ 0.05; **p ≤ 0.01; ***p ≤ 0.001; ****p ≤ 0.0001).

**FIGURE 2:**
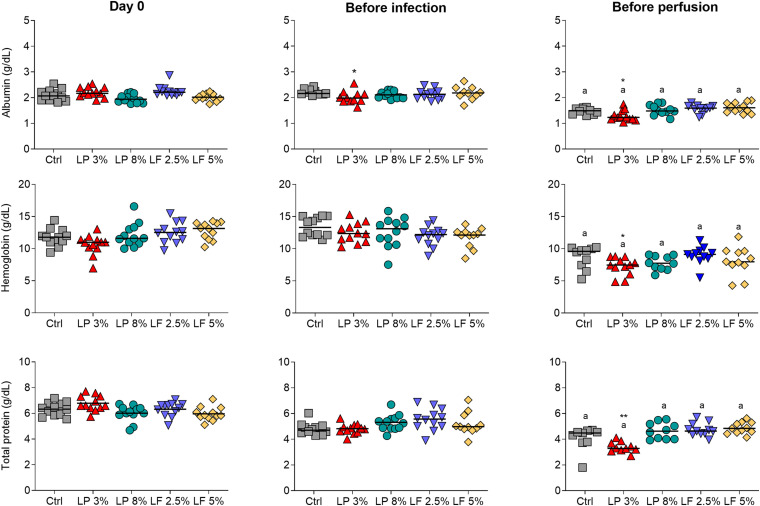
Serum concentrations of albumin, hemoglobin and total protein in mice fed with different experimental diets. Mice received different restricted diets: Control (Ctrl), Low protein 3 and 8% (LP 3% and LP 8%, respectively) and Low fat 2.5 and 5% (LF 2.5% and LF 5%, respectively). Albumin, hemoglobin and total protein levels (from top to bottom) were measured on the first day of the experiment (day 0), before infection with ∼100 cercariae of *S. mansoni* (∼day 50) and before perfusion of the hepatic portal system (∼day 100). The median is shown on each graph. Results are representative of two independent experiments (*n* = 10–12 mice per group). Significant differences were determined by Kruskal-Wallis test followed by Dunn’s multiple comparison test. “*” denote differences between LP 3% and Ctrl groups (*p ≤ 0.05; **p ≤ 0.01); “a” denote differences between before infection and before perfusion in each group.

Body weight, concentration of hemoglobin in whole blood, and concentration of total protein and albumin in serum, were measured in animals from Control (Ctrl) and experimental groups (LP 3% and LP 8%, LF 2.5% and 5%) to verify malnutrition status. One week after initiation of the experimental diets, LP 3% mice exhibited significant decrease in body weight compared to Ctrl mice (Figure 1A). Significant differences in body weight were maintained between these two groups up to the end of the experimental period. In contrast, animals fed with other experimental diets showed no significant difference in body mass, in comparison to the Ctrl group. Seven weeks after initiation of the experimental diets, mice were infected with ∼100 cercariae of *S. mansoni*, as indicated by the dashed line in Figure 1A. Body mass was not further changed by infection (Figure 1A). However, after infection, LP 3%-fed mice showed reduced consumption of food (Figure 1B) and lower protein intake (Figure 1C), compared to the Ctrl group. LP 8%-fed mice did not show any change in food intake, but presented a significant reduction in protein intake that reflects the protein deficiency of the diet. Furthermore, before infection, a significant reduction in albumin concentration was observed in LP 3% mice (Figure 2). Significant differences were not observed before infection in total protein and hemoglobin levels among mice from the different experimental groups. However, infection with *S. mansoni* triggered a decrease in albumin, total protein and hemoglobin levels in LP 3% mice compared to control mice, and in all groups when compared to the levels observed before infection (Figure 2). An additional experiment was performed including uninfected control groups (4 different groups of mice in total: uninfected Ctrl and LP 3%, and infected Ctrl and LP 3%), in order to evaluate whether the infection and/or the diet affect systemic levels of these biomarkers. Before infection, seventy days after initiating the experimental diet, both the uninfected and infected LP 3% groups showed a significant reduction in the albumin concentration in relation to mice fed with the control diets (Supplementary Figure 1). The impact of the LP 3% on the hemoglobin levels was observed 120 days after starting the diets. Lower levels of albumin and hemoglobin were observed in infected mice regardless of the diet. Infection only reduced total serum protein levels in LP 3%-fed mice. These data demonstrate that the diet with 3% protein in its composition alone caused malnutrition in mice, which is then aggravated by infection with *S. mansoni.*

### Low Protein 3% Diet Impacts *S. mansoni* Infection With Reductions in Eggs Trapped in the Liver and Parasite Fecundity

**FIGURE 3:**
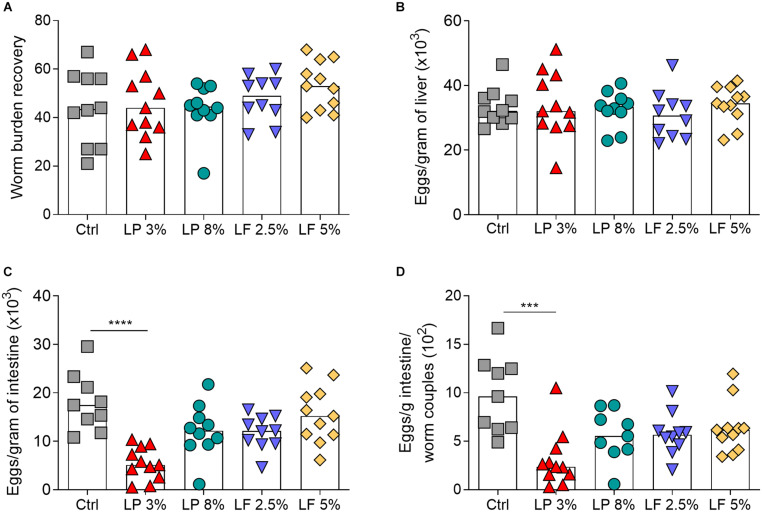
Parasitological parameters of mice fed with different experimental diets. Mice received different restricted diets: Control (Ctrl), Low protein 3 and 8% (LP 3% and LP 8%, respectively) and Low fat 2.5 and 5% (LF 2.5% and LF 5%, respectively). Adult worms were recovered approximately 50 days post-infection **(A)**. The numbers of eggs per gram of liver **(B)** and intestine **(C)** were determined by optical microscopy after digestion of both tissues with 10% KOH. Fecundity was determined by the ratio between the number of eggs per gram of intestine and the number *S. mansoni* couples recovered by perfusion **(D)**. Bars represent the median number of worms recovered or eggs per gram of organ. Results are representative of two independent experiments (*n* = 10–12 mice per group). Significant differences were determined by Kruskal-Wallis test followed by Dunn’s multiple comparison test (***p ≤ 0.001; ****p ≤ 0.0001).

In order to evaluate the impact of the different diets on *S. mansoni* infection, mice from all experimental groups were challenged with ∼100 cercariae and the parasite burden was determined 50 days later. No differences were observed in the adult worm burden (Figure 3A) recovered, and in the number of eggs trapped in the liver, of mice fed with different experimental diets and those from the control group (Figure 3B). However, the number of eggs trapped in the intestine was significantly lower in LP 3% mice than in control mice (Figure 3C). To assess whether this reduction occurred due to changes in parasite fecundity, the ratio between the number of eggs per gram of intestine and the number of adult worm pairs was calculated. As expected, LP 3% mice exhibited a significant reduction in parasite fecundity in comparison to the control group (Figure 3D).

### Low Protein 3% Diet Affects Liver Pathology Induced by *S. mansoni* Infection

**FIGURE 4:**
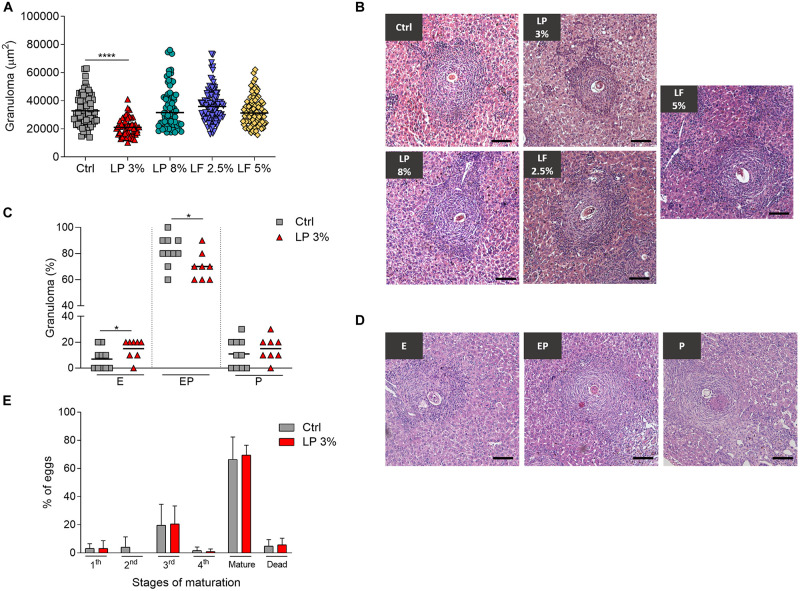
Hepatic granuloma area in mice fed with different experimental diets. Mice received different restricted diets: Control (Ctrl), Low protein 3 and 8% (LP 3% and LP 8%, respectively) and Low fat 2.5 and 5% (LF 2.5% and LF 5%, respectively). At 50 days post-infection, sections of liver from each mouse were obtained and slides were prepared and stained with hematoxyline-eosine. Approximately 100 granulomas containing a single well-defined egg at the exudative-productive stage were randomly selected and measured from the livers of mice from each experimental group. The total area of the granulomas was expressed in square micrometers (μm^2^) and represented as the median **(A)**. Representative histological sections of liver granulomas from each experimental group **(B)**. Frequencies of granulomas in Ctrl and LP 3% groups **(C)** and representative types of granulomas: E, exudative; EP, exudative-productive; P, productive **(D)**. Percentage of *S. mansoni* eggs in different stages of development in the ileum: immature (first, second, third or fourth stages), mature and dead **(E)**. Results are representative of two independent experiments (*n* = 10–12 mice per group). Significant differences were determined by Kruskal-Wallis test followed by Dunn’s multiple comparison test **(A)**, Student’s *t*-tests **(C)** or Two-way ANOVA followed by Sidak’s multiple comparison test **(E)** (*p ≤ 0.05; ****p ≤ 0.0001). Scale bar = 100 μm (100x).

LP 3% mice showed a significant reduction in the hepatic granuloma area in comparison the Ctrl group (Figures 4A,B). In order to explore the impact of malnutrition on granuloma development, we examined the developmental stages of hepatic granulomas. At 50 days after infection, the liver of the *S. mansoni* infected Ctrl and LP 3% groups exhibited the expected occurrence of granulomas in different stages of maturation: Exudative (E), Exudative-productive (EP) and Productive (P). However, LP 3% mice showed an increase in the frequency of the E and decrease in the EP stages compared to the Ctrl group (Figures 4C,D). In order to assess whether *S. mansoni* egg maturation was impairing granuloma formation an oogram analysis was performed, but no significant difference in the percentage of the eggs in different maturation stages was observed between Ctrl and LP 3% groups (Figure 4E).

### Cytokine Levels Triggered by *S. mansoni* Were Affected by Nutrient Composition of the Diet

**FIGURE 5:**
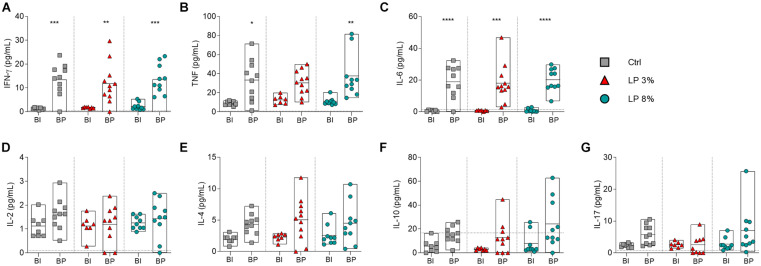
Cytokine profile of mice fed with different experimental diets. Mice received different restricted diets: Control (Ctrl), Low protein 3 and 8% (LP 3% and LP 8%, respectively). Serum samples were obtained before infection (∼day 50), and before perfusion (∼day 100) for cytokine measurement. Levels of IFN-γ **(A)**, TNF **(B)**, IL-6 **(C)**, IL-2 **(D)**, IL-4 **(E)**, IL-10 **(F)**, and IL-17 **(G)** production were measured using the CBA Th1/Th2/Th17 kit. BI: before infection; BP: before perfusion. Results are representative of two independent experiments (*n* = 6–11 mice per group) and significant differences were determined by one-way ANOVA followed by Holm-Sidak’s multiple comparison test (*p ≤ 0.05; **p ≤ 0.01; ***p ≤ 0.001; ****p ≤ 0.0001).

**FIGURE 6:**
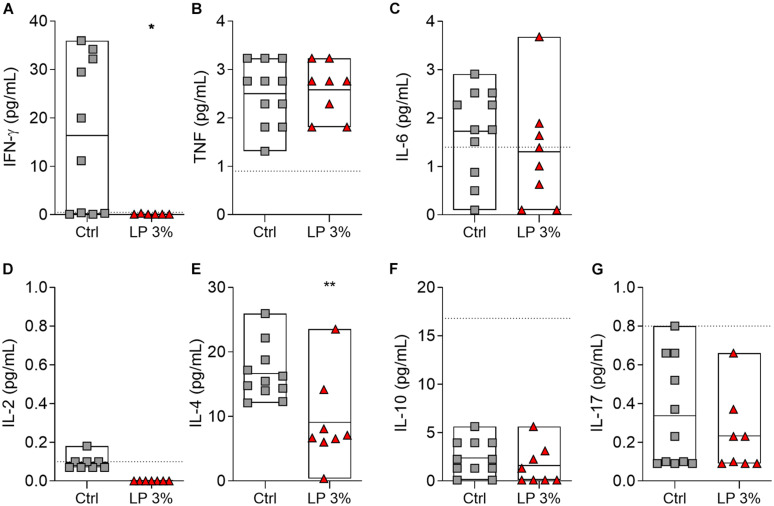
Cytokine profile in the liver of mice fed with different experimental diets. Mice received Control (Ctrl) or Low protein 3% (LP 3%) diet and liver homogenates were obtained 50 days after infection (before perfusion) for cytokine measurement. Levels of IFN-γ **(A)**, TNF **(B)**, IL-6 **(C)**, IL-2 **(D)**, IL-4 **(E)**, IL-10 **(F)**, and IL-17 **(G)** were measured using the CBA Th1/Th2/Th17 kit, *n* = 8–11 mice per group. Significant differences were determined by Student’s *t*-tests or Mann-Whitney test (*p ≤ 0.05; **p ≤ 0.01).

The levels of circulating cytokines in serum from the mice of all experimental groups were determined on day zero (before starting the experimental diets) (Supplementary Figure 2), before *S. mansoni* infection (BI) and before perfusion (BP) (Figure 5). Levels of cytokines were similar among groups at all time points evaluated (Supplementary Figure 2 and Figure 5). The levels of IL-2, IL-4, IL10, and IL-17 were also not significantly altered by infection (Figures 5D–G). Regardless of the diet, increased levels of IFN-γ and IL-6 were observed in mice BP when compared to BI (Figures 5A,C). On the other hand, induction of TNF upon infection was observed in Ctrl and LP 8% animals, but the same was not noticed in LP 3%-fed mice (Figure 5B). In addition, LP 3% mice showed decreased levels of cytokines IL-4 and IFN-γ in liver homogenate 50 days after infection by *S. mansoni* compared to the Ctrl group (Figure 6).

## Discussion

The relationship between nutrition, specifically protein deficiency, and infections has been evaluated previously (Magalhães et al., 1986; Rezende et al., 1997a; Anstead et al., 2003; Taylor et al., 2013; Losada-Barragán et al., 2019). However, the impact of malnutrition on susceptibility to *S. mansoni* infection is not well understood. In this study, we established a murine model of malnutrition to study the impact of this condition on experimental *S. mansoni* infection. We used diets with restricted protein (3 or 8%) or lipid (2.5 or 5%) content based on the formulations proposed by American Institute of Nutrition (AIN) (Reeves et al., 1993). AIN formulations are widely used in a variety of studies with different types of approaches (Malafaia et al., 2009; Lemes et al., 2016; Souza et al., 2020). These formulations provide researchers a definition for maintaining consistency between studies, as well as enabling specific individual nutrient adjustment to meet different goals (Pellizzon and Ricci, 2020).

In our model, we employed the most widely used protein concentration range (2–8%) (12, 23, 24). Mice fed with LP 3% diet, in contrast to those fed with LP 8%, exhibited weight loss and, therefore, malnutrition. Data published by others demonstrate malnutrition even in mice fed with diets containing 8% protein. The different outcome observed in our study can be attributed to differences in the food composition, as previous studies used more restricted diets with multi-deficiency in macronutrients (mainly proteins and lipids), as well as minerals and vitamins (Teodósio et al., 1990; Coutinho et al., 1992; Rezende et al., 1997a; Barros et al., 2014; Santos et al., 2020).

As previously reported, in our study, besides presenting weight loss, animals fed with the LP 3% diet consumed less food, and, therefore, less protein than control mice (Borelli et al., 2007; Fock et al., 2010). Possible mechanisms related to anorexia may be explained by hypothalamic modifications or alterations in the serum concentration of branched amino acids, which may modulate the effects of neurotransmitters, leading to a possible adaptive process in order to prevent important changes in nitrogen balance (Gietzen, 1993; Fock et al., 2007).

In addition, mice fed with the LP 3% diet showed a significant reduction in serum albumin levels in the period before infection and in all biochemical biomarkers assessed before perfusion. Although there are many factors that influence levels of serum proteins in plasma (Cabrerizo et al., 2015), albumin, hemoglobin and total protein are commonly used as biochemical indicators of the nutritional status (Barros et al., 2014; Nakajima et al., 2014; Oliveira et al., 2014; Santos et al., 2016; Zhang et al., 2017). Serum proteins, such as albumin, are characterized as negative acute-phase proteins, and their levels are affected by a number of inflammatory illnesses, mainly those associated with liver function (Fleck, 1989; Bharadwaj et al., 2016). Thus, in our model, both malnutrition and infection by *S. mansoni* affected serum albumin levels. Another parameter altered by the infection of animals fed with the LP 3% diet was the hemoglobin concentration. Among the possible mechanisms responsible for the anemia reported in hosts infected by parasites of the genus *Schistosoma*, the most common cause is iron deficiency due to extra-corporeal loss (elimination of gastrointestinal blood and feces) (Mahmood, 1966; Farid et al., 1967), splenic sequestration (Mahmoud and Woodruff, 1973; Friis et al., 2003) or anemia developing upon inflammation (Mwatha et al., 1998; Abdel-Aaty et al., 1999; Means, 2000). It is worth mentioning that long periods of LP 3% diet also decrease the levels of hemoglobin. Together, these data demonstrate that only the LP 3% diet was able to induce malnutrition in mice characterized by the decrease in body mass, serum albumin, hemoglobin levels and food consumption, all indicators of a nutritional deficiency.

Previous studies have demonstrated the impact of host malnutrition on the biology and differentiation of *S. mansoni*, inducing phenotypic modifications in adult worms of both sexes (Neves et al., 2002; Oliveira et al., 2003). We did not find a significant difference in the number of adult worms recovered in malnourished animals compared to controls. However, there is no consensus in the literature regarding the impact of malnutrition on the establishment of infection (Simões et al., 2002; Coutinho et al., 2007; Barros et al., 2014). These divergences may be due to experimental approaches, such as mouse lineage, diet compositions and/or time of infection.

Mice fed with LP 3% diet have lower numbers of eggs trapped in the intestine. A high metabolism appears to be necessary for an adequate oogenesis by *S. mansoni* adult females (Davies et al., 2001). In this regard, animals fed with LP 3% showed a reduction in parasite fecundity, but no impact on egg maturation. Morphological alterations of the schistosome reproductive system may explain the lower oviposition of worms recovered from malnourished experimental hosts (Oliveira et al., 2003; Blank et al., 2006). Moreover, these changes have been attributed to the reduced availability of essential nutrients necessary for adequate growth and development of the parasite in the blood vessels (Neves et al., 2002). Parasite growth and development are also dependent on immune factors (Amiri et al., 1992; Davies et al., 2001; Blank et al., 2006). TNF is required for oviposition *in vivo* and *in vitro* (Amiri et al., 1992). Our data showed that at day 50 of infection with *S. mansoni* increased circulating IFN-γ, IL-6 and TNF levels were observed in mice with adequate nutritional status. In contrast, mice fed with LP 3% diet did not display increased production of TNF after *S. mansoni* infection, suggesting that the different cytokine profile observed in malnourished mice in response to *S. mansoni* infection influence oviposition. However, further experiments are necessary to clarify the mechanisms responsible for the reduced parasite fecundity observed in these mice.

In our study, mice fed with low fat diets showed no changes in adult worm burden and in the number of eggs trapped in tissues. Previous studies have shown that *S. mansoni* cannot synthesize lipids *de novo*, and, therefore, the incorporation by the parasites of lipids from their environment is intense (Meyer et al., 1970; Brouwers et al., 1997). *In vitro* experiments have shown that several classes of lipids are incorporated more-or-less efficiently by adult worms, and there is interconversion from one class of lipid to another (Rumjanek and Simpson, 1980). Furthermore, the absence of fatty acid and cholesterol synthesis by *S. mansoni* indicate that there are efficient mechanisms for capturing host lipids (Rumjanek et al., 1988). In fact, when exposed to human serum, the parasites increase the expression of lipoprotein (LDL) receptors on the surface of their tegument (Rumjanek et al., 1988; Xu and Caulfield, 1992).

Hepatic granulomas are dynamic structures that show variable morphology depending on several factors, such as immune cells, cytokine patterns (Silva et al., 2000) and host nutritional status (Coutinho et al., 2007; Rezende et al., 1997a). Previous investigations on the relationships between schistosomiasis and host nutritional status have demonstrated that mice infected by *S. mansoni* and fed with a low-protein diet develop, in the acute phase of the disease, smaller peri-ovular granulomas with reduced hepatic collagen deposition (Coutinho et al., 2007; Barros et al., 2014).

Studies assessing the pathogenesis of schistosomiasis demonstrate the role of CD4^+^ T cells in the formation of hepatic granulomas. The Th1 response triggered by schistosomula and adult worm antigens play a role in the initial stage of granuloma formation (Pearce and MacDonald, 2002; Wynn et al., 2004; Wilson et al., 2007). IFN-γ is important in the protective mechanism against hepatic fibrosis, while TNF can aggravate the pathogenesis (Henri et al., 2002; Booth et al., 2004), due to its roles in activating hepatic stellate cells and collagen deposition (Pradere et al., 2013; Yang and Seki, 2015). Th2 cytokines also have an important role in fibrogenesis, as demonstrated by the fact that mice deficient in STAT6 have granulomas with smaller area and less collagen content (Kaplan et al., 1998). Furthermore, IL-4 and IL-13 promote the differentiation of alternatively activated macrophages, which are known to enhance collagen production through an arginase-1-dependent mechanism (Hesse et al., 2001). On the other hand, IL-10 has a regulatory role in schistosomiasis, preventing the development of excessive pathologies mediated by both Th1 and Th2 responses (Hoffmann et al., 2000).

At the tissue level, LP 3% mice show significantly reduced levels of IFN-γ and IL-4 in the liver. Moreover, LP 3% mice had smaller granulomas when compared with the Ctrl group. Studies demonstrated that in the absence of IFN-γ signaling and Th2 cytokines smaller hepatic granulomas are observed during *S. mansoni* infection (Rezende et al., 1997b; Kaplan et al., 1998; Jankovid et al., 1999). Thus, the reduced levels of these cytokines could have influenced granuloma size in malnourished mice. Additionally, TNF and IL-13 have been associated with fibrosis (Tarrats et al., 2012; Schwartz et al., 2014). Our data showed no increase in circulating TNF levels in LP 3% mice upon infection, and, although we have not measured IL-13 levels, studies demonstrated that the level of this cytokine is lower in the absence of IL-4 (Cheever et al., 1994). Our data also showed, for the first time, a delay in granuloma development due to nutritional status, in which in the livers of LP 3%-fed mice there is an increased percentage of granulomas in the exudative phase and a decreased percentage in the exudative-productive phase. This delay in the granuloma development, and their reduced area, observed in the LP 3% mice, cannot be attributed to an impairment of egg maturation. However, further studies are still needed to investigate if protein-deficient diets impact the expression and secretion of egg antigens, such as IPSE and Omega-1 (Schramm et al., 2007; Everts et al., 2009), which are described to be involved in driving the Th2 response.

Overall, our results demonstrate that the 3% low-protein diet was able to establish malnutrition in mice that influenced the outcome of experimental *S. mansoni* infection, reducing egg burden in the intestine, parasite fecundity and granuloma size. Additionally, malnutrition leads to a delay in granuloma development. These parameters may be associated with the cytokine milieu found in the liver. The establishment of this murine malnutrition model will enable future studies aiming to better understand the complex relationship between malnutrition, infection, and immunity. Additionally, this model could be used to test the effectiveness of any vaccine against diseases which affect populations living with food insecurity.

## Data Availability Statement

The original contributions presented in the study are included in the article/Supplementary Material, further inquiries can be directed to the corresponding author/s.

## Ethics Statement

The animal study was reviewed and approved by the Ethics Committee of Animal Use of FIOCRUZ. Licence number: LW2/18.

## Author Contributions

CF, LA, and PM designed the research, performed the data analysis, and wrote the manuscript. CF, LA, PM, and RG performed the experiments and discussed the data. All authors contributed to the article and approved the submitted version.

## Conflict of Interest

The authors declare that the research was conducted in the absence of any commercial or financial relationships that could be construed as a potential conflict of interest.
