# Supplementary figures and images for: Schistosoma mansoni Infection Is Impacted by Malnutrition
Source: Front Microbiol. 2021 Mar 19;12:635843. doi: 10.3389/fmicb.2021.635843 (PMC8017134; doi:10.3389/fmicb.2021.635843)

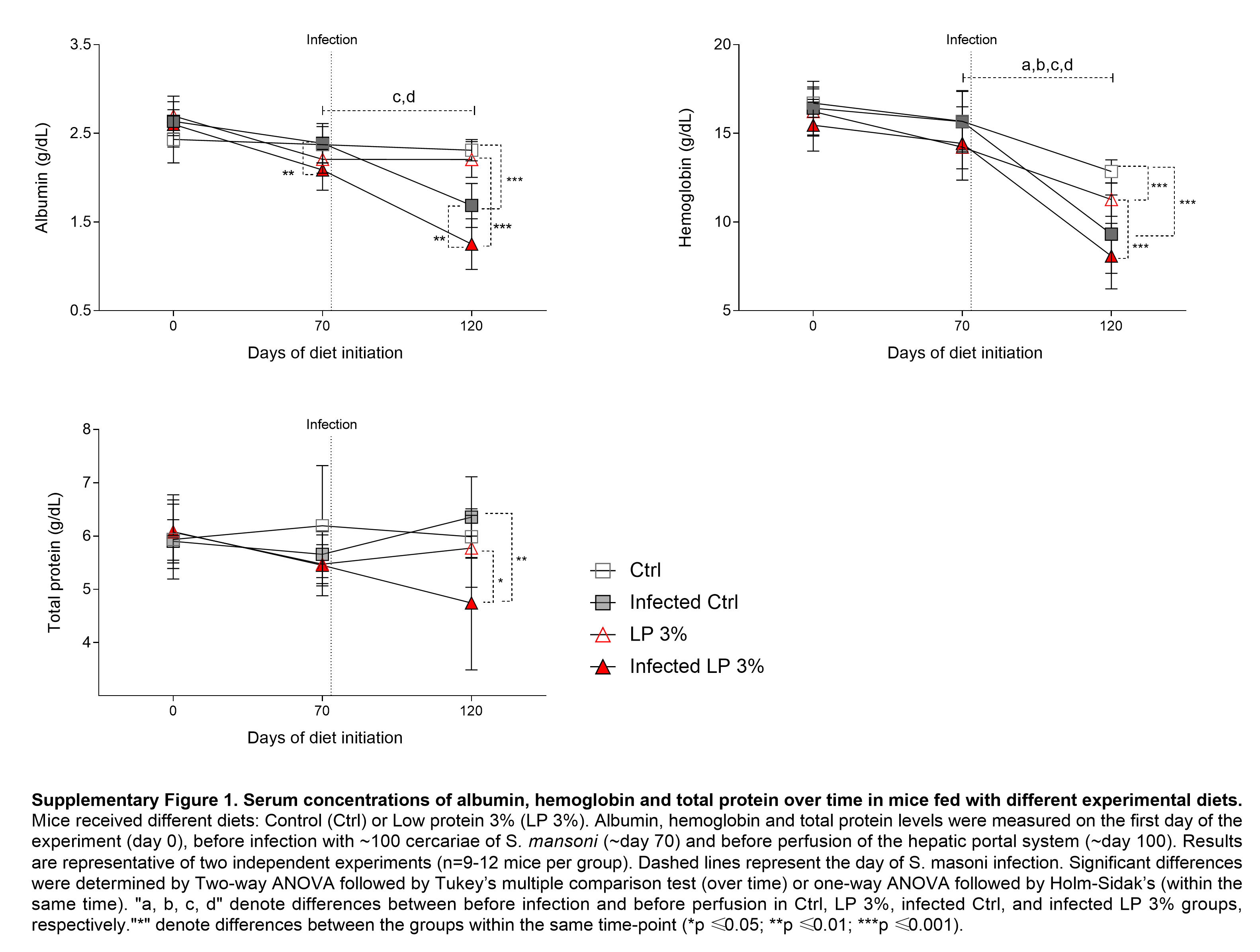

Supplement: Supplementary file 1 [file Image_1.TIF]

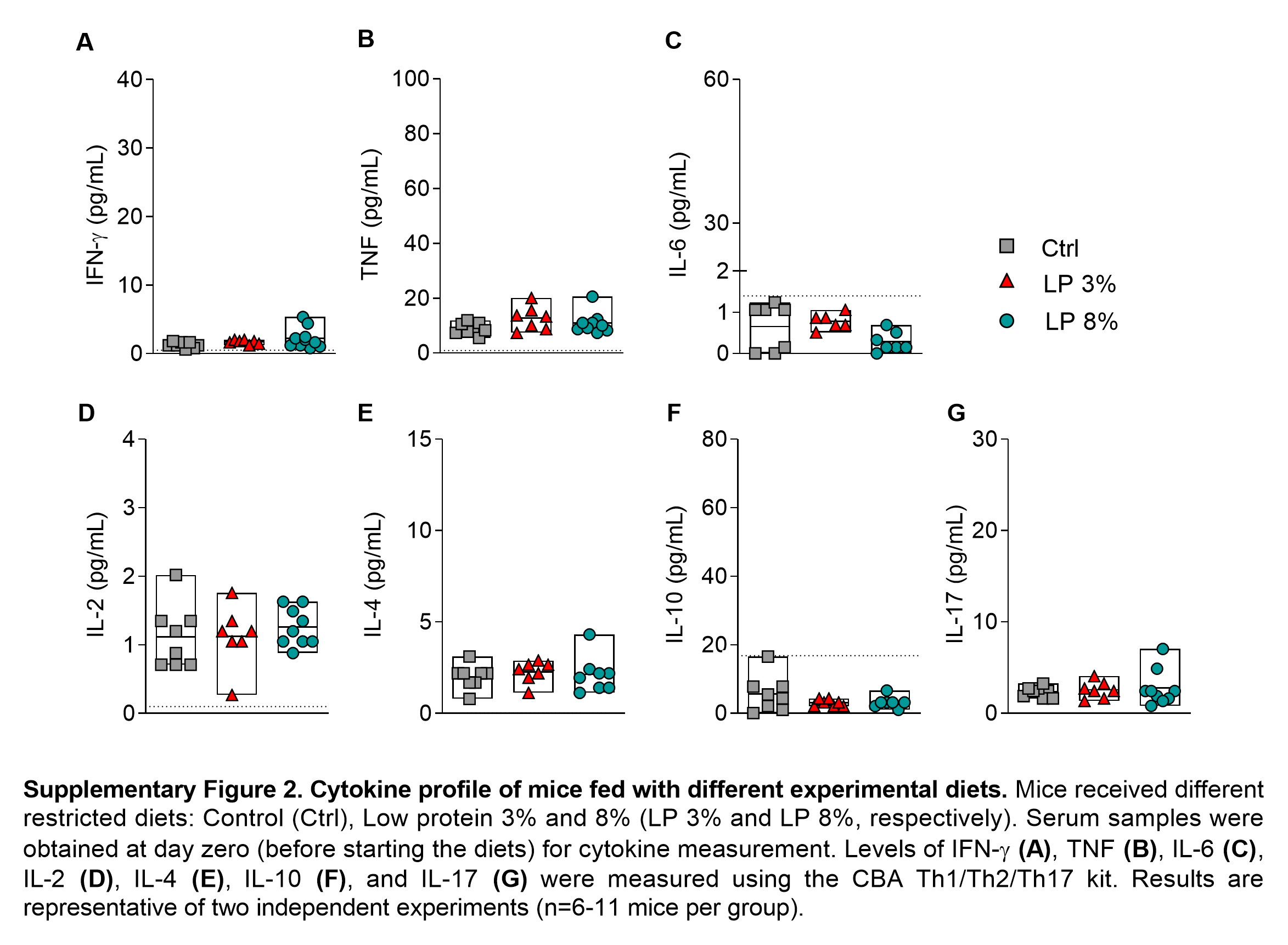

Supplement: Supplementary file 2 [file Image_2.TIF]
